# Editorial: Heterodienes in organic synthesis

**DOI:** 10.3389/fchem.2024.1403024

**Published:** 2024-04-08

**Authors:** Alexey Yu. Sukhorukov

**Affiliations:** Laboratory of Organic and Metal-Organic Nitrogen-Oxygen Systems, N. D. Zelinsky Institute of Organic Chemistry, Moscow, Russia

**Keywords:** heterodienes, vinylogous systems, Michael addition, cycloadditions, annulations, cascade reactions, N-heterocycles, bioorthogonal chemistry

## Introduction

Vinylogous systems have always been in focus of organic chemists due to their unique reactivity, structure, and synthetic application (Curti et al., 2020). Heterodienes are among the most simple and valuable vinylogous systems in organic chemistry. The presence of heteroatoms in the conjugated diene induces specific polarization of the π-system leading to versatile reactivity patterns (Lopes et al., 2018).

**FIGURE 1:**
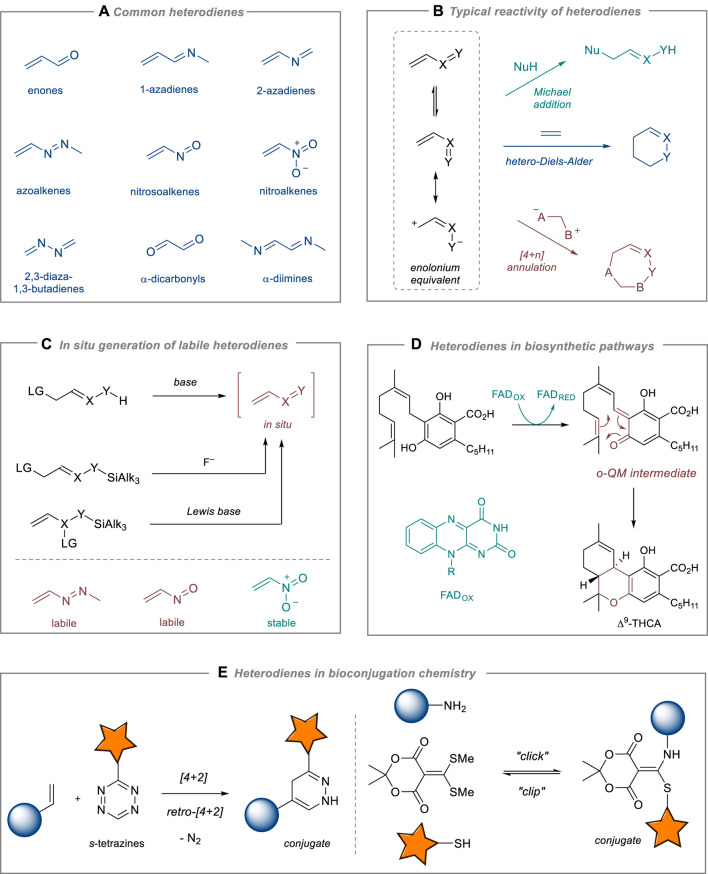
Chemistry and applications of heterodienes. **(A)** Common heterodienes. **(B)** Typical reactivity of heterodienes. **(C)** In situ generation of labile heterodienes. **(D)** Heterodienes in biosynthetic pathways. **(E)** Heterodienes in bioconjugation chemistry.

Among the most widely utilized heterodienes in organic synthesis are α,β-unsaturated carbonyl compounds (enones), 1- and 2-azadienes, 1,2-diaza-1,3-butadienes (azoalkenes), nitro- and nitrosoalkenes, 2,3-diaza-1,3-butadienes, and α-dicarbonyl compounds and α-diimines (Figure 1A). Although they are chemically distinct species, their reactivity shares common features (Figure 1B). First, most of heterodienes are reactive Michael acceptors in reactions with various nucleophiles (Lopes et al., 2018; Weinreb, 2019). Second, similarly to normal dienes, heterodienes enter [4 + 2]-cycloaddition reactions. Due to their electron-deficient nature, heterodienes react only with electron-rich dienophiles via an inverse-electron demand Diels–Alder reaction (Baiazitov and Denmark, 2013; Png et al., 2017). Third, being highly polarized 1,4-synthons, heterodienes are convenient partners for stepwise [4 + 1]- [4 + 3]- and [4 + 4]- and other [4 + n]-annulation processes involving ylides, carbenoids, and related species (Selvaraj et al., 2020; Ushakov et al., 2022; Wang et al., 2024). Additionally, they are commonly involved in multi-component condensation reactions that lead to the formation of valuable heterocyclic scaffolds (Attanasi et al., 2009; Lopes et al., 2018; Heredia-Moya et al., 2022). Heterodiene reactions can be conducted using a variety of organo- and metal-based catalysts, enabling the asymmetric synthesis of valuable products, especially those found in natural sources and pharmaceuticals. Enantioselective Michael addition, hetero-Diels-Alder, and cascade reactions with stable heterodienes (mostly, conjugated enones and nitroalkenes) have been successfully developed in recent years (Jiang and Wang, 2013).

The Frontiers Research Topic “Heterodienes in Organic Synthesis” comprises a Research Topic of original research articles dealing with the chemistry and applications of heterodienes. This Research Topic consists of four articles, which reflect on modern trends in the synthetic chemistry of the azoalkenes, nitrosoalkenes, as well as α,β-unsaturated carbonyl compounds and imines.

## Stability of heterodienes

Heterodienes are known to be reactive and chemically labile species. Thus, azoalkenes and nitrosoalkenes, unless stabilized with bulky or strong EWG groups, are prone to dimerization and polymerization reactions. These heterodienes are generated *in situ* from the corresponding stable precursors (α-halohydrazones, α-halooximes and their silyl ethers, ene-nitroso acetals, Figure 1C). In contrast, conjugated nitroalkenes are normally bench-stable, yet highly reactive heterodienes. Michael addition to nitroalkenes affords β-functionalized nitro derivatives that can be further transformed into amines (via reduction of NO_2_ group), carbonyls (via Nef reaction), oximes (via interrupted Nef and Meyer reactions), and other useful products (Ballini et al., 2007; Sukhorukov, 2023). Nitroalkenes are recognized for their stability and versatile chemistry, making them essential building blocks in organic synthesis along with enones (Halimehjani et al., 2014).

## Heterodienes in biosynthesis

Apart from organic synthesis, heterodienes play a crucial role in the fields of biochemistry and biotechnology, with continuously expanding applications. Recent research on the biosynthesis of natural compounds has shown that Nature extensively exploits the versatile chemistry of heterodienes. The biosynthetic machinery utilizes the conjugate addition of enolate-type nucleophiles to α,β-unsaturated carbonyl compounds to synthesize structurally diverse natural products, for example, polyketides (Miyanaga, 2019). More surprisingly, the hetero-Diels-Alder reaction of unstable *ortho*-quinone methides (*o*-QMs) catalyzed by specific enzymes (in particular, hetero-Diels-Alderases) was recently discovered to be a key stage in the biosynthesis of cannabinoids (Purdy et al., 2022) and some sesquiterpenes (Chen et al., 2019) (Figure 1D). Heterodiene chemistry offers extensive possibilities for bioconjugation via fast and catalyst-free “click”-like reactions compatible with *in vivo* conditions, for example, [4 + 2]-cycloaddition of 1,2,4,5-tetrazines (*s*-tetrazines) (Oliveira et al., 2017; Zare et al., 2022). Moreover, a reversible character of the Michael addition to heterodienes has been utilized to design “clip” reactions for controllable reversible bioconjugation chemistry (Diehl et al., 2016) (Figure 1E).

## Azoalkenes

Conjugated azoalkenes are highly promising intermediates in organic synthesis since they are synthetic equivalents of enolonium cation (reversely polarized synthon to enolate anion) (Attanasi et al., 2009; Uteuliyev et al., 2015). Being powerful Michael acceptors, azoalkenes react with a variety of nucleophiles leading to α-substituted hydrazones that can be further hydrolyzed to ketones. However, the use of *P*-nucleophiles in these reactions is very limited. The report by Alexey Sukhorukov et al. describes a convenient protocol for the Michael addition of phosphine oxides R_2_P(O)H to the *in situ*-generated azoalkenes. The developed method provides a convenient route to β-hydrazonophosphine oxides that are precursors to important organophosphorus compounds, including phosphorylated *N*-heterocycles, α-aminophosphonates, and vinylphosphonates.

## Nitroso- and nitroalkenes

Nitroso- and nitroalkenes are extensively utilized as 4π synthons in hetero-Diels-Alder reactions with electron-rich alkenes. This methodology provides straightforward access to 1,2-oxazines and their *N*-oxides (cyclic nitronic esters) that serve as intermediates in the synthesis of highly functionalized natural products with multiple stereogenic centers (Denmark et al., 2008; Malykhin et al., 2024). The report by Teresa Pinho e Melo et al. deals with experimental and theoretical studies on the regioselectivity of the [4 + 2]-cycloaddition of ethyl nitrosoacrylate with pyrroles, indoles, and 1,6-dihydropyrrolo[3,2-*c*]carbazoles leading to fuzed 1,2-oxazine systems. Using the developed approach, a new heterocyclic system, namely, hexahydropyrido[4′,3':4,5]pyrrolo[3,2-c]carbazole, was assembled by the authors.

## Other heterodienes

Multi-component one-pot reactions using heterodienes are currently undergoing significant development. In this Research Topic, Yue Zhang et al. report new photocatalytic trichloromethyl radical-triggered annulative reactions of amide-linked 1,7-diynes with polyhalomethanes. This process involves a cascade of Kharasch-type addition/nucleophilic substitution/elimination reactions leading to densely substituted polyhalogenated quinolin-2(1H)-one derivatives. In another report in this field, Fabiana Nador et al. developed a Cu-catalyzed A3-type coupling between pyridine-2-carbaldehyde, an aromatic alkyne, and a substituted tetrahydroisoquinoline to give new indolizine-dihydroisoquinoline hybrid dyes. The obtained products exhibit pH-dependent changes in the UV-Vis spectra and color which makes them attractive candidates to use as pH indicators.

## Conclusion

The cutting-edge research articles published in this Frontiers Research Topic highlight that the chemistry of heterodienes continues to be an exciting and challenging research area, in which many more discoveries will be made.
